# Effects of Icariin on Modulating Gut Microbiota and Regulating Metabolite Alterations to Prevent Bone Loss in Ovariectomized Rat Model

**DOI:** 10.3389/fendo.2022.874849

**Published:** 2022-03-24

**Authors:** Shanshan Wang, Shengjie Wang, Xiaoning Wang, Yunteng Xu, Xin Zhang, Yidan Han, Hui Yan, Linglong Liu, Lili Wang, Hongzhi Ye, Xihai Li

**Affiliations:** ^1^ College of Integrative Medicine, Fujian University of Traditional Chinese Medicine, Fuzhou, China; ^2^ Academy of Integrative Medicine, Fujian University of Traditional Chinese Medicine, Fuzhou, China; ^3^ Key Laboratory of Fujian University of Traditional Chinese Medicine, Fuzhou, China; ^4^ Basic Discipline Laboratory of Integrative Medicine, Fujian University of Traditional Chinese Medicine, Fuzhou, China

**Keywords:** icariin, postmenopausal osteoporosis, gut microbiota, metabolic pathway, bile acid, amino acid, fatty acid

## Abstract

Postmenopausal osteoporosis (PMOP) is an estrogen deficiency-induced bone loss, which has been shown an association with an altered gut microbiota (GM). Gut microbiota-bone axis has been recognized as a crucial mediator for bone homeostasis. Icariin (ICA) is an effective agent to delay bone loss by regulating the bone homeostasis. Thus, we hypothesize that ICA can prevent bone loss by modulating GM and regulating metabolite alterations. The effects of ICA on bone metabolism improvement in ovariectomized (OVX) rats and their relationships with the GM and fecal metabolites were investigated. Micro-computed tomography (micro-CT) and hematoxylin-eosin (HE) staining showed a typical bone boss in OVX group, while ICA or estradiol (E2) administration exhibited positive effects on bone micro-architecture improvement. The GM such as Actinobacteria, Gammaproteobacteria, Erysipelotrichi, Erysipelotrichales, Enterobacteriales, Actinomycetales, *Ruminococcus* and *Oscillospira* significantly correlated to serum bone Gla-protein (BGP), receptor activator of nuclear factor-κB (RANK), receptor activator of nuclear factor-κB ligand (RANKL), osteoprotegerin (OPG) and tartrate resistant acid phosphatase (TRACP). Further *t*-test revealed a substantial variation of the GM and fecal metabolites in different treatments. Among them, *Lachnoclostridium*, *Butyricimonas*, *Rikenella*, *Paraprevolla*, *Adlercreutzia*, *Enterorhabdus*, *Anaerovorax*, *Allobaculum*, *Elusimicrobium*, *Lactococcus*, *Globicatella* and *Lactobacillus* were probably the key microbial communities driving the change of bile acid, amino acid and fatty acid, thereby leading to an improvement of PMOP. The significant up-regulation of L-Saccharopine, 1-Aminocyclohexadieneacid and linoleic acid after ICA administration suggested important contributions of amino acid and fatty acid metabolisms in the prevention and treatment of PMOP. Taken together, our study has provided new perspectives to better understand the effects of ICA on PMOP improvement by regulating GM and the associated fecal metabolites. Our findings contribute to develop ICA as a potential therapy for PMOP.

## Introduction

Postmenopausal osteoporosis (PMOP) is a worldwide disease often observed in the ageing woman. A high prevalence and a lack of effective therapy have made it the leading cause of disease burden (1). The pathophysiological mechanisms underlying PMOP are complicated and not fully understood. Estrogen as well as other medicines including bisphosphonates, denosumab and parathyroid hormone (PTH) analogs are popular choices for the prevention and treatment of PMOP (2–5). However, several undesirable side effects of the above drugs on women’s health limit their wide and long-term administration (2, 6). It is still urgent to find more alternative choices to prevent and treat PMOP.

Icariin (ICA), 2-(4′-methoxyphenyl)-3-rhamnoside-5-hydroxyl-7-glucoside-8-(3′-methyl-2-butylene)-4-chromanone, as the main active flavonoid ingredient of Herba Epimedii, has been widely used in treating bone and joint diseases (7). Although there is insufficient clinical evidence, ICA has shown great efficacy for the treatment of PMOP in many studies (8, 9). ICA administration increased bone mineral density (BMD) and serum osteoprotegerin (OPG), but decreased serum bone Gla-protein (BGP) concentration in ovariectomized (OVX) rats (10). Receptor activator of nuclear factor-κB (RANK) and receptor activator of nuclear factor-κB ligand (RANKL) play vital roles in the collapse of cartilage and subchondral bone, while the result can be inhibited by ICA (11). Also, ICA has shown ability in decreasing the serum levels of tartrate-resistant acid phosphatase (TRACP) and consequent reducing the bone resorption (12). These findings suggest that ICA has potential drug target for treating PMOP.

So far, several possible mechanisms underlying bone loss improvement under ICA administration have been reported. The mostly-studied regulation mechanism towards PMOP is to accelerate bone formation through Wnt/β-catenin signaling pathway (10, 13, 14). This signaling pathway often co-functions with bone morphogenetic proteins (BMPs) or ERα to promote osteogenic differentiation under ICA treatment (14, 15). Recently, others potential regulating mechanisms such as the enhancement of IGF-I coupled non-genomic ERα signaling (16), the promotion of signal transducer and activator of transcription (STAT) (17) and the stimulation of mitogen-activated protein kinase (MAPK) (11) have also been reported. Noticeably, both MAPK and Wnt/β-catenin signaling pathways are involved in the IL-1β-induced expression of OPG, RANKL and RANK (11). This indicates that anti-inflammation is an important process in ICA-induced PMOP improvement, which can also be regulated by IGF-I signaling pathway (16, 18).

Another power driving the PMOP improvement is gut microbiota (GM) alteration. The roles of GM-bone axis in PMOP regulation have been increasingly noticed (19). It was reported that a suppression of bone loss with the administration of *Agastache rugosa* extract was regarded to be associated with the induction of osteoblast differentiation due to the alteration of GM (20). The significant correlations between GM and serum RANK, OPG and TRACP or inflammatory factors suggested a potential role of GM-bone axis in improving PMOP (21). More and more studies have also shown an influence of the altered GM on gut homeostasis *via* regulating bile acid, amino acid and fatty acid metabolisms, thereby leading to an improvement of PMOP (22–24). Correspondingly, those GM capable of bile acid, amino acid and fatty acid metabolisms were already used to prevent PMOP in OVX model (18, 24). It is apparent that ICA can modulate GM to improve the intestinal barrier function through regulating p38 MAPK and NF-κB signaling pathway or inhibiting intestinal inflammation (25–27). As such, we hypothesize that the therapeutic effects of ICA on PMOP may be closely associated with GM-bone axis regulation.

In present study, we employed OVX rat as a model to evaluate the effects of ICA on PMOP improvement at GM-bone axis. The following validations were implemented: 1) the effects of ICA administration on bone micro-architecture and serum physiological indexes, 2) the composition and abundance of GM and their correlations with serum biomarkers and 3) the relationships between GM and fecal metabolites and their potential roles in improving PMOP. We believe that the results have provided compelling evidence of ICA in regulating GM-bone axis that may contribute to the development of ICA as a novel therapy for PMOP.

## Materials and Methods

### Ethical Approval

All the animal experiments were performed in accordance with the Guide for the Care and Use of Laboratory Animals published by the US National Institutes of Health (NIH Publication, 8^th^ edition, 2011). Before experiment, the principles and guidelines were also approved by the Laboratory Animal Welfare and Ethics Committee of Fujian University of Traditional Chinese Medicine ((Min) SYXK 2019-0007).

### Experimental Animals and Dietary Treatments

Sixty 2-months old female specific-pathogen-free (SPF) Sprague-Dawley (SD) rats weighting from 180 to 220 g were purchased from Shanghai Laboratory Animal Co. Ltd. (Shanghai, China; animal license NO. SCXK (Hu) 2017-0005). The rats were allowed to acclimate to animal room for 1 week before experiment. The acclimated animals were randomly divided into 4 groups, including control group (Sham, n = 15), ovariectomy group (OVX, n = 15), ovariectomy plus ICA administration group (OVX+ICA, n = 15) and ovariectomy plus estradiol (E2) administration group (OVX+E2, n = 15). The OVX rats were obtained according to Jiang et al. (6) with a slight modification. Briefly, the rats were anesthetized with pentobarbital sodium (100 mg/kg i.p.) and the ovaries were removed to establish PMOP model. After surgery, the established rats were intramuscularly injected penicillin (200,000 units per rat) for 3 days to prevent infection and housed in individual cage. As to Sham group, a given amount of adipose tissue around ovary was removed to mimic the ovariectomy.

A commercial rodent diet and water were provided during the whole period of 12 weeks. Icariin or E2 at a concentration of 20 mg/kg/d or 0.1 mg/kg/d dissolved in 0.9% saline was administered intragastrically to animals in experimental groups every day. In contrast, the animals in Sham and OVX groups were administered 0.9% saline with the same dose of 0.5 mL as OVX+ICA and OVX+E2 groups. Animals in all groups were housed in SPF room at a temperature of 22-26°C and a relative humidity of 40%-70% with 12 h/12 h light/night cycle in the Laboratory Animal Center of Fujian University of Traditional Chinese Medicine.

### Microtomographic Observation and Histomorphometric Analysis of Lumbar

After the last administration, the rats were fasted for 12 h. Live rats were scanned on micro-computed tomography (micro-CT; Guangzhou Zhongke Kesheng Medical Technology Co., LTD) to examine the structure and morphology of the lumbar. The micro-CT scan indexes were set at a voxel resolution of 20 μm, a beam angle increment of 0.72°, an X-ray voltage of 70 kV, an X-ray power of 7 W, a current intensity of 100 μA and an exposure time of 100 ms for a total of scanning time of 10 min. For each run, 3 groups of bones and a calibration phantom were analyzed in pairs to standardize the grayscale values and maintain the consistency of all samples. The calibrated three-dimensional (3D) images were reconstructed by MedProject (28). The typical histomorphometric indexes, i.e., trabecular bone mineral content (BMD) and bone volume fraction (BV/TV), were analyzed by ZZKS-Micro-CT4.1. More details about the assessed regions of interest (ROI) and the growth plate reference slice for ROI selection can be obtained from Fu et al. (29).

After micro-CT scanning, the rats were euthanized and the lumbar tissues were separated carefully. The obtained lumbar was fixed with 4% paraformaldehyde for 24 h, followed by thrice wash in Milli-Q water for 1 min per run and decalcified in 10% ethylenediaminetetraacetic acid (EDTA) for 8 weeks. A fresh EDTA was added to the system to renew the solution every two days. Before slicing, the decalcified lumbar was dehydrated by ethyl alcohol at the concentrations of 70%, 80%, 90%, 95% (4 h per run) and 100% (thrice, 20 min, 30 min and 70 min per run), transparentized twice by xylene (15 min and 30 min per run), waxed thrice (20 min by soft paraffin (two runs) and 40 min by hard paraffin), and finally embedded by paraffin as required (30). The embedded lumbar was sectioned to a 4-μm thick slice on a RM2135 Slicer (Leica, Germany). After dewaxing, the hydrated slices were stained with hematoxylin eosin (HE) for histomorphometric analysis (29).

### Serum Biochemical Analysis

All venous blood was collected from the abdominal aorta of the rats under anesthesia and stored in a sterile vacuum tube. A total of 5 mL of blood were stayed statically for 4-5 h at room temperature and then centrifuged at 4°C, 3500 r/min for 15 min (Eppendorf 5430R, Germany). The serum in supernatant was transferred to a new tube and stored at -80°C before use. The biochemical indexes including bone Gla-protein (BGP), OPG, RANK, RANKL and TRACP were tested by enzyme-linked immunosorbent assay (ELISA) according to the manufacturer’s instructions (31).

### Gut Microbiota Analysis Based on High-Throughput Sequencing

One hundred mg of the fresh feces were collected and stored in sterile containers under -80°C until further analysis. Bacterial genomic DNA was extracted by using a FastDNA^®^ SPIN Kit for Soil (MP Biomedicals, USA), while DNA concentrations and quality were determined by a Nanodrop^®^ ND-1000 UV-vis spectrophotometer (NanoDrop technologies, Wilmington, DE, USA) and 1.0% agarose gel electrophoresis. For each treatment, 10 samples were used for high-throughput sequencing. All obtained samples were diluted to 1 ng/μL with sterile Milli-Q water.

The V3-V4 variable region of 16S rRNA were amplified using the barcoded primers 5’-ACTCCTACGGGAGCAGCA-3’ (27F) and 5’-GGACTACHVGGGTWTCTAAT-3’ (1492R). The amplification system of polymerase chain reaction (PCR) contained 30 ng of sample DNA, 1 μL of each of the 5 μM primer, 12.5 μL of 2 × Taq Plus Master Mix, 3 μL of 2 ng μL^−1^ Bovine Serum Albumin (BSA) and 7.5 μL of sterile Milli-Q water. The PCR programs consisted of a total of 30 cycles including 2 min at 95°C, 20 s at 95°C, 30 s at 55°C and 30 s at 72°C, and a final extension for 5 min at 72°C (32). The amplicons were purified and recovered by a AxyPrepDNA Gel Extraction Kit (AXYGEN), followed by a library construction by using NEB Next^®^Ultra™DNA Library Prep Kit for Illumina (NEB, USA) following manufacturer’s instructions. The library quality was assessed on a Qubit2.0 Fluorometer (Thermo Scientific) and Agilent Bioanalyzer 2100 system. Finally, the established amplicons were pooled in equimolar proportion and paired-end sequenced on Illumina HiSeq2500 platform.

The paired-end reads of original DNA fragments were merged using Flash (v1.20) and Pear (v0.96) according to the overlap of the reverse complementary sequences. After removing the chimera and short or low-quality reads, the established sequences were analyzed by Uparse software package using the Uparse-OTU and Uparse-OTUref algorithms. In short, the sequences with ≥ 97% similarity were assigned to a same OTU. Following, the clear data were submitted to NCBI-SRA database with the accession number PRJNA807063. In our study, the representative sequences for each OTU were picked to annotate their taxonomic information by using RDP Classifier algorithm. To evaluate the change of GM among treatments, the community distribution and relative abundance of GM were taxonomically analyzed from the levels of phylum to species. However, only those microbial communities ranking top 10 of the relative abundance were used for further analysis. Moreover, the potential GM involved in PMOP improvement were screened by correlating them to serum biomarkers BGP, OPG, RANK, RANKL and TRACP based on Spearman analysis (SPSS, version 21). Furthermore, the significant up-regulation and down-regulation of the GM at different taxonomic levels between Sham and OVX, OVX+ICA and OVX, OVX+E2 and OVX were also fully evaluated. Statistical analysis was performed by *t*-test at *p* = 0.05 using GraphPad Prism for Windows (Version 6.01, USA). Finally, the key phylotypes of GM in each treatment were identified by Linear Discriminant Analysis (LDA) Effect Size (LefSe) analysis with both LDA value calculation and cladogram construction (33).

### Fecal Metabolite Analysis

The fresh feces (~100 mg) were mixed thoroughly with 100 μL sterile Milli-Q water (vortical vibration for 1 min) and 100 μL methanol-acetonitrile solution (v/v = 1:1) (vortical vibration for 1 min), respectively, and ultrasonically treated for 1 h at 4°C. The mixture was stayed statically for 1 h to precipitate the proteins at -20°C and centrifuged for 20 min at 4°C and 14000 r/min. The obtained supernatants were subsequently freeze-dried (FreeZone 6 plus, Labconco, USA) for 48 h and stored at -80°C until further use. Before analysis, each sample was re-dissolved by 100 μL methanol-acetonitrile-water solution (v/v/v = 2:2:1) with 5 min of vortical vibration and centrifuged again for 20 min at 4°C and 14000 r/min.

Liquid chromatography coupled with mass spectrometry/mass spectrometry (LC-MS/MS) analysis was performed on an Agilent 1290 Infinity LC (Agilent, USA) with an AB Triple TOF 5600/6600 MS system (AB SCIEX, USA). An ACQUITY UPLC BEH Amide C18 column (1.7 μm, 2.1 mm × 100 mm; Waters, UK) and an ACQUITY UPLC HSS T3 column (1.8 μm, 2.1 mm × 100 mm; Waters, UK) were used for the reversed phase separation. The column oven was maintained at 25°C. The flow rate of LC was 0.3 mL/min and the mobile phase consisted of solvent A (water + 25 mM ammonium acetate + 25 mM ammonium hydroxide) and solvent B (acetonitrile). Gradient elution conditions were set as follows: 0-0.5 min, 95% B; 0.5-7 min, linear decrease of B from 95% to 65%; 7-8 min, linear decrease of B from 65% to 40%; 8-9 min, 40% B; 9-9.5 min, linear increase of B from 40% to 95%; 9.1-12 min, 95% B. The injection volume for each sample was 3 μL. The samples in autosampler were stayed at 4°C in the whole analysis process.

Both positive (POS) and negative (NEG) modes of electrospray ionization (ESI) were used for the detection of fecal metabolites. The ESI conditions for Agilent 5600 system were gas temperature of 250°C, drying gas of 16 L/min, nebulizer of 20 psig, sheath gas temperature of 400°C, sheath gas flow of 12 L/min, Vcap of 3000 V, nozzle voltage of 0 V. Other conditions include fragment of 175 V, mass range of 50-1200, acquisition rate of 4 Hz and cycle time of 250 ms. The obtained ions were further identified by AB Triple TOF 6600 system with ion source gas1 of 40 and gas2 of 80, curtain gas of 30, source temperature of 650°C, IonSapary voltage floating of ±5000 V (POS and NEG modes). As to secondary ion MS (SIMS or MS2) analysis, the data were obtained by using information dependent acquisition (IDA; exclude isotopes within 4 Da, candidate ions to monitor per cycle: 10) in high sensitivity mode with de-clustering potential of ±60 V (POS and NEG modes), and collision energy of 35 ± 15 eV. Data collection was performed according to mass range of 50-300, 290-600, 590-900 and 890-1200 (four times for each internal) to increase the spectrum collection efficiency. The structure of the collected ions was identified by MetDDA and LipDDA developed by Shanghai Applied Protein Technology Co. Ltd (Shanghai, China).

Univariate analysis and multivariate analysis were used to identify the potential key metabolites. While the fold change (FC) analysis can identify the differential metabolites between treatments, *t*-test helps to verify whether a significant difference is present. The variable importance for the projection (VIP) obtained from orthogonal partial least squares discriminant analysis (OPLS-DA) model, along with FC and *p*-value, is also an important index for evaluating the differences among treatments. In our study, the metabolites screened by VIP > 1, FC > 1.2 (up-regulation) or < 0.8333 (down-regulation) and *p*-value < 0.05 were regarded as the significant differential candidates. To have a full evaluation of the annotated metabolites and their differences in expression patterns in different samples, we used the qualitative expression levels of the significant differential metabolites for hierarchical clustering analysis of each group.

### Correlation Analysis Between GM and Fecal Metabolites

To evaluate whether can gut microbes influence bone metabolisms through changing the compositions of fecal metabolites, the relationships between the relative abundance of significant differential GM in genus level (*t*-test; *p*-value < 0.05) and the significant differential metabolites (VIP > 1 and *p*-value < 0.05) were correlatively analyzed. The analyses included correlation coefficient calculation (Spearman), Spearman correlation analysis-based network mapping (Cytoscape 3.5.1) and Spearman correlation analysis-based hierarchical clustering heat mapping (R 3.4.2 Heatmap package). It was considered that there was a significant relationship between GM and fecal metabolites when *p*-value was < 0.05. Finally, each paired GM and fecal metabolite with significant correlation was fully compared with previous studies to evaluate their potential roles in PMOP prevention and (or) treatment under ICA administration.

### Statistical Analysis

All the experiments were repeated independently at least three times. The results are presented as the mean ± standard deviation (SD). R package and SPSS 21.0 (IBM, USA) were used to perform Spearman and *t*-test analyses as required. Significant differences were calculated according to one-way analysis of variance (ANOVA) by Tukey’s multiple comparisons test at *p* = 0.05 using GraphPad Prism for Windows (Version 6.01, USA) unless otherwise noted.

## Results

### Verification of Therapeutic Effects of ICA on PMOP

**Figure 1:**
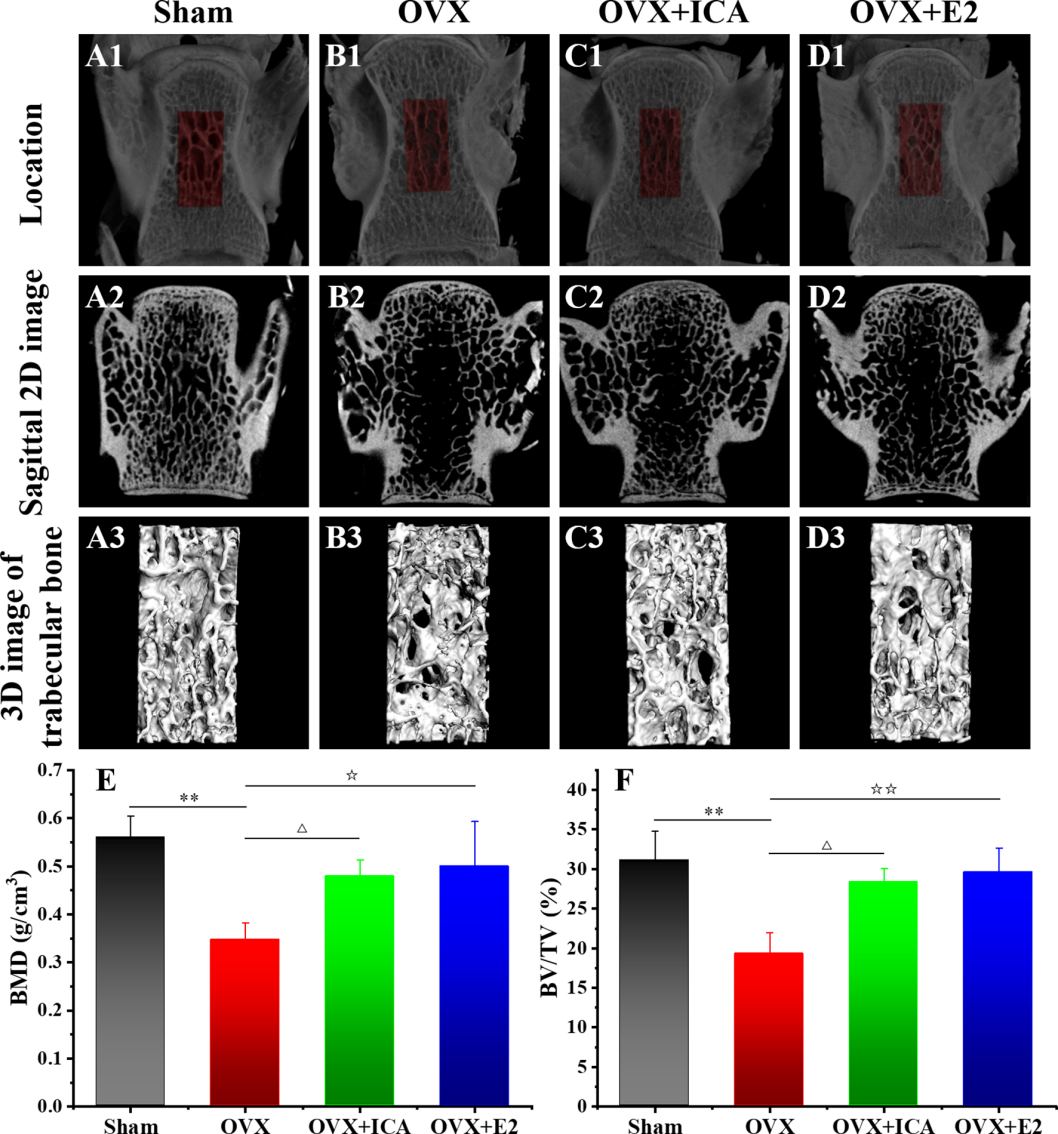
Therapeutic effects of different treatments on the bone histomorphometry of PMOP model. Micro-CT images of the bone structure for Sham **(A1–A3)**, OVX **(B1–B3)**, OVX+ICA **(C1–C3)** and OVX+E2 **(D1–D3)** (n = 6). Morphometric analysis of BMD **(E)** and BV/TV **(F)** in local area (three samples per group at each time point). ^△,☆^ *p <* 0.05, ^☆☆^, **p < 0.01.

Micro-CT images show that the bone trabecular of SD rats in Sham group is of close arrangement, regular shape, uniform thickness and consistent direction (**Figures 1A1–A3**). The ovariectomy can lead to an obvious loss of the bone trabecula, which becomes thinner with a fractured structure, an irregular arrangement and a wider spacing, thereby forming cavities in the located area (**Figure 1B1–B3**). Either ICA or E2 administration exhibited positive effects on improving the trabecular micro-architecture (**Figures 1C1–C3, D1–D3**). Both BMD and BV/TV in local area of the lumbar vertebra also verified a significant bone loss in OVX rats (*p <* 0.01), while ICA and E2 administration significant increased BMD by 38% (*p <* 0.05) and 44% (*p <* 0.05) and BV/TV by 47% (*p <* 0.05) and 53% (*p <* 0.01), respectively (**Figures 1E, F**). These results showed that ICA prevented estrogen deficiency-induced PMOP in OVX rats.

**Figure 2:**
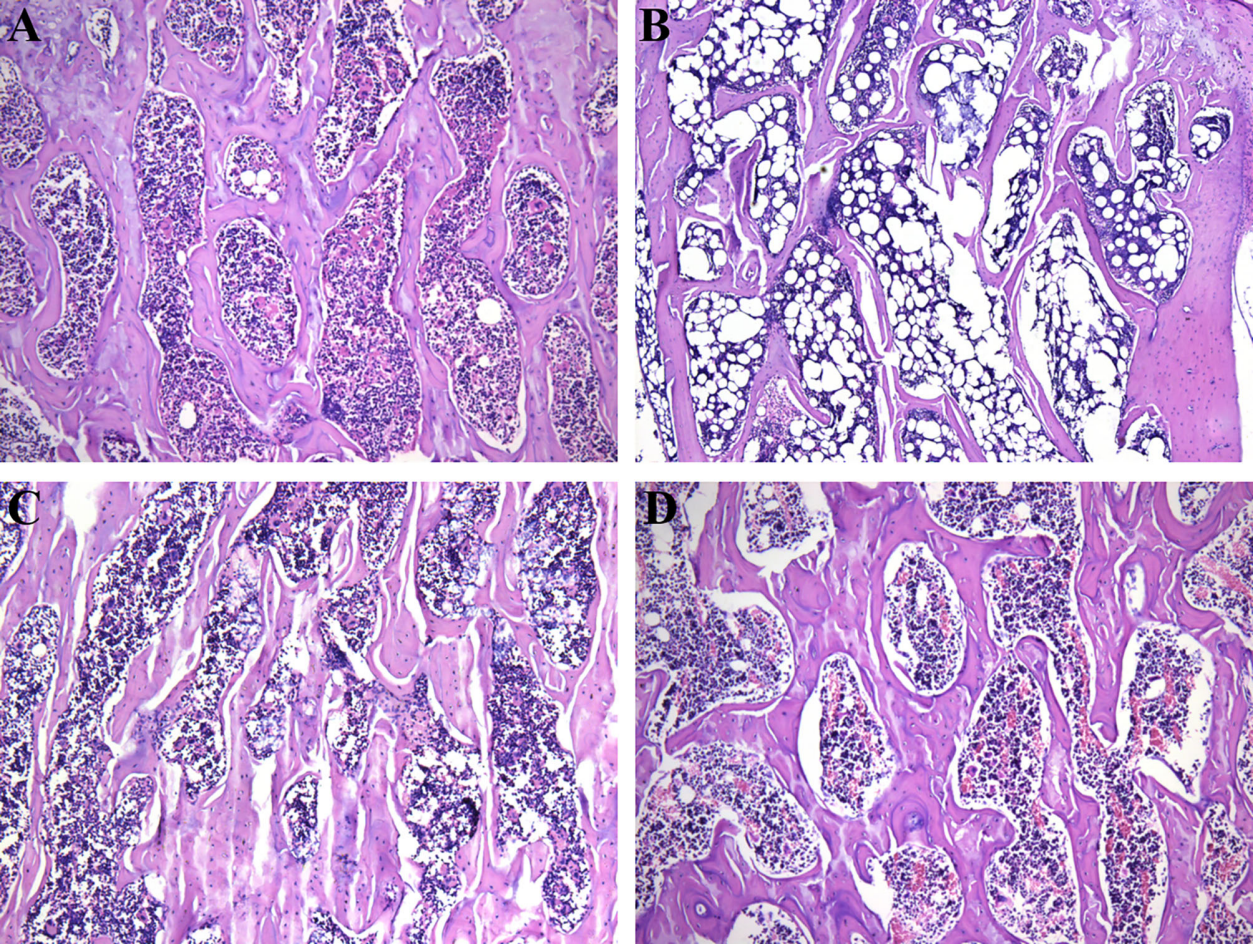
Therapeutic effects of different treatments on the bone trabecular structure (100 ×) of PMOP model. **(A-D)** represent the HE images of Sham, OVX, OVX+ICA and OVX+E2 groups, respectively.

Besides *in situ* micro-CT observation, a HE staining was also performed in this study to evaluate the bone trabecular structure in different treatments. **Figure 2** shows a histology structure of the fifth lumbar vertebra stained by HE. As expected, a regular mesh network of the bone trabecula was observed in Sham group (**Figure 2A**). However, a typical trabecular structure was missing, instead a considerable structure change and emerging adipocytes in marrow cavity were found in OVX group (**Figure 2B**). The HE staining also showed us a clear improvement of the bone trabecula in PMOP rats administrated with ICA or E2 (**Figure 2C**). These results verified a potential role of ICA, served as an estrogen-like substitution, in improving the estrogen-deficient symptoms.

### Regulation of ICA on Serum Biomarkers Associated With Bone Metabolisms

**Figure 3:**
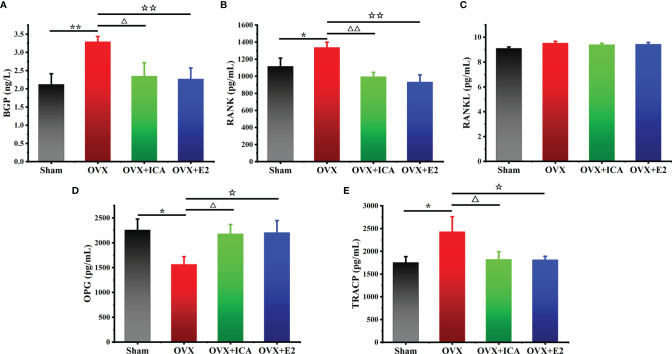
Therapeutic effects of different treatments on serum concentrations of BGP **(A)**, OPG **(B)**, RANK **(C)**, RANKL **(D)** and TRACP **(E)** in PMOP model (n = 6). ^*,△,☆^ *p <* 0.05, ^**,△△,☆☆^ *p <* 0.01.

Compared to Sham group, the serum BGP in OVX rats significantly increased from 2.1 to 3.3 ng/L (*p <* 0.01) (**Figure 3A**). When administrated with ICA, serum BGP concentration decreased to 2.4 ng/L (*p <* 0.05), close to those observed in Sham and OVX+E2 groups (**Figure 3A**). The serum RANK concentrations in Sham, OVX, OVX+ICA and OVX+E2 followed a similar trend to BGP, being up to 1339 pg/mL in OVX group and 935-1117 pg/mL in other treatments (**Figure 3B**). However, both ovariectomy and ICA or E2 administration did not significant change the concentration of serum RANKL (**Figure 3C**), a biomarker that can be bound to RANK to enhance osteoclastogenesis (34). Unlike RANKL, OPG, as another ligand of RANK, in the serum of OVX rats was significantly down-regulated to 1572 pg/mL by 31% lower than Sham group (*p <* 0.05) (**Figure 3D**). Either ICA or E2 administration improved the dysfunction of ovary, resulting in a significant up-regulation of serum OPG concentration (*p <* 0.01) (**Figure 3D**). Besides, we also determined the serum TRACP concentration and found an increase of TRACP by 38% in OVX group (*p <* 0.05) and a similar concentration in OVX+ICA and OVX+E2 as compared to Sham group (**Figure 3E**). These results showed that ICA administration was of great ability to influence bone metabolisms *via* regulating serum BGP, RANK, RANKL, OPG and TRACP to a desirable concentration.

### Verification of GM-Bone Axis Targets Involved in ICA-Mediated Improvement of PMOP

**Figure 4:**
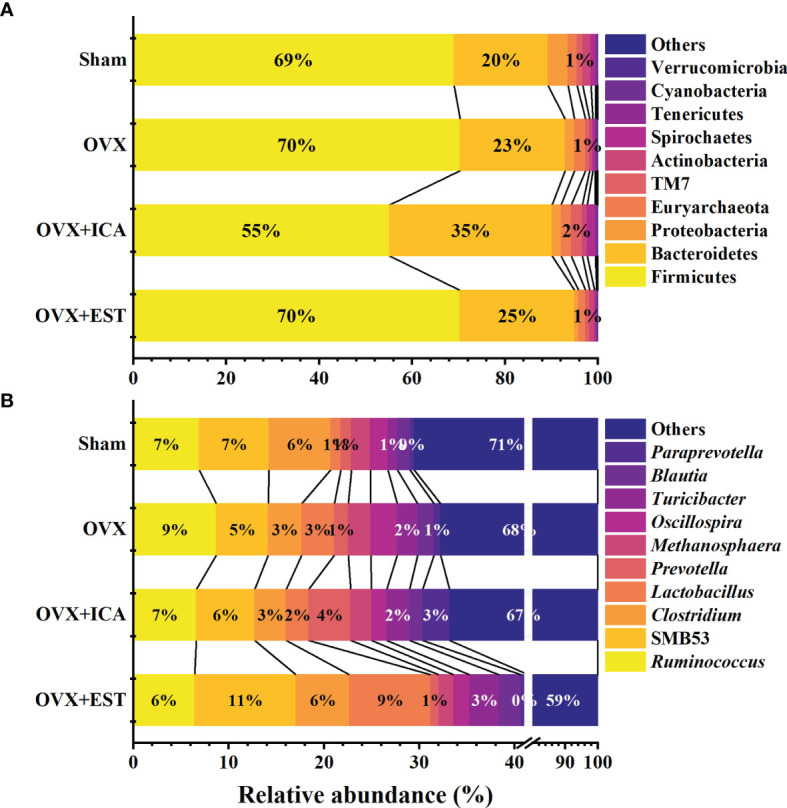
Therapeutic effects of different treatments on the relative abundance of GM at the taxonomic levels of phylum **(A)** and genus **(B)** (n = 10). Only the Latin manes of the top 10 GM were given, while the low abundant ones were put together and shown as ‘‘Others’’.

In phylum level, Firmicutes and Bacteroidetes predominated in all treatments, being the lowest abundance of Firmicutes by 55% and the highest abundance of Bacteroidetes by 35% both observed in OVX+ICA group (**Figure 4A**). These phylum communities in Sham, OVX and OVX+E2 groups were 69%-70% and 20%-25%, respectively (**Figure 4A**). In other taxonomic levels, it was apparent that the dominant GM belonging to Firmicutes included the genera *Ruminococcus*, *Clostridium*, *Lactobacillus*, *Oscillospira* and SMB53 group, the families Ruminococcaceae, Clostridiaceae, Lachnospiraceae, Lactobacillaceae and S24-7 group, the orders Clostridiales and Lactobacillales, and the classes Clostridia and Bacilli (**Figure 4B**; **Supplementary Figure S1**). As to those GM belonging to Bacteroidetes, the genera *Prevotella* and *Paraprevotella* in Prevotellaceae, a family belonging to the order of Bacteroidales in the class of Bacteroidia, were likely to be dominant in rat gut (**Figure 4B**; **Supplementary Figure S1**). Statistical analysis suggested that the dominant GM mainly be positively correlated with RANK and TRACP and negatively correlated with RANKL and OPG (**Table 1**). The most eye-catching genera were *Ruminococcus* and *Oscillospira*, which probably contributed to the significant up-regulation of RANK and TRACP and down-regulation of OPG (**Table 1**). It was also worth noting that some nondominant GM belonging to Actinobacteria, Gammaproteobacteria, Erysipelotrichi, Enterobacteriales and Acetinomycetales were important in regulating serum RANK and RANKL concentrations (**Table 1**).

**Table 1:** Pearson analysis of the correlations between GM and serum biomarkers probably associated with bone metabolisms.

GM^a^		BGP	RANK	RANKL	OPG	TRACP
p_Actinobacteria	Pearson’s r	-0.750	-0.267	-0.989 ^*b^	0.700	-0.700
	*p*-value	0.250	0.733	0.011	0.300	0.300
c_Gammaproteobacteria	Pearson’s r	0.744	0.951 ^*^	0.089	-0.770	0.761
	*p*-value	0.256	0.049	0.911	0.230	0.239
c_Erysipelotrichi	Pearson’s r	0.629	0.952^*^	-0.116	-0.676	0.671
	*p*-value	0.371	0.048	0.884	0.324	0.329
o_Enterobacteriales	Pearson’s r	0.867	0.995**^c^	0.254	-0.900	0.899
	*p*-value	0.133	0.005	0.746	0.100	0.101
o_Erysipelotrichales	Pearson’s r	0.629	0.952 ^*^	-0.116	-0.676	0.671
	*p*-value	0.371	0.048	0.884	0.324	0.329
o_Actinomycetales	Pearson’s r	-0.878	-0.472	-0.952 ^*^	0.841	-0.841
	*p*-value	0.122	0.528	0.0480	0.159	0.159
g_*Ruminococcus*	Pearson’s r	0.941	0.970 ^*^	0.418	-0.962 ^*^	0.961 ^*^
	*p*-value	0.059	0.030	0.582	0.038	0.039
g_*Oscillospira*	Pearson’s r	0.924	0.954*	0.409	-0.951 ^*^	0.953 ^*^
	*p*-value	0.076	0.0460	0.591	0.0490	0.0470

a p, phylu; c, class; o, order; g, genus.

b * Significant correlation at p-value of 0.05 based on Two-tailed test.

c ** Significant correlation at p-value of 0.01 based on Two-tailed test.

**Figure 5:**
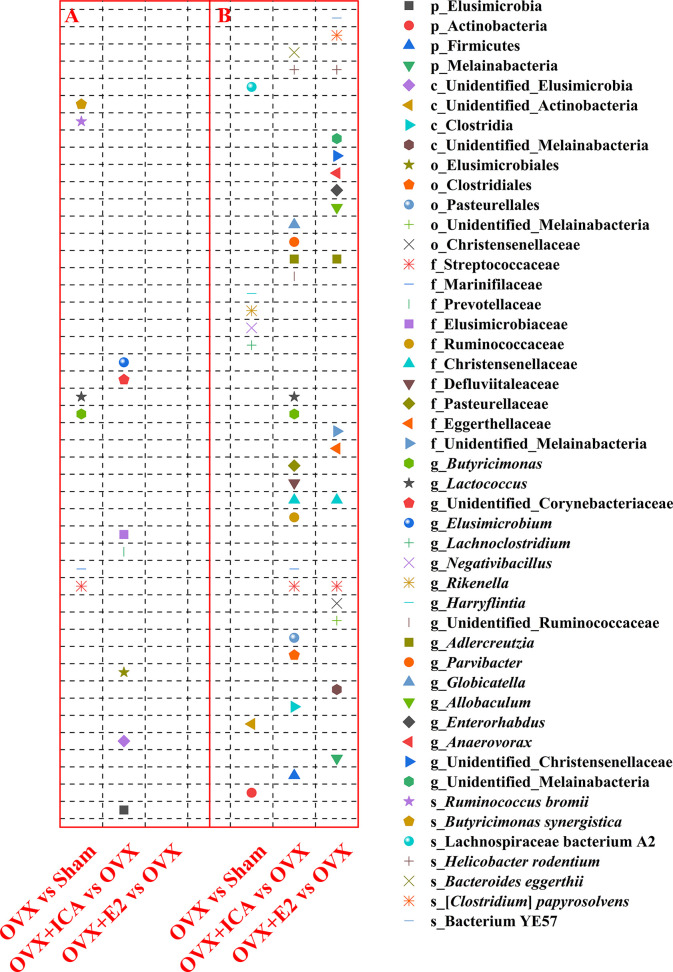
Therapeutic effects of different treatments on the GM at different taxonomic levels by significant up-regulation **(A)** and down-regulation **(B)** between paired treatments (n = 10). The statistical analysis was performed by *t*-test at *p* = 0.05 using GraphPad Prism for Windows (Version 6.01, USA).

Among treatments, a total of 4 plyla, 4 classes, 5 orders, 10 families, 17 genera and 7 species varied significantly based on *t*-test at *p* = 0.05 (**Figure 5**). Specifically, the families Streptococcaceae and Marinifilaceae, the genera *Butyricimonas* and *Lactococcus* and the species *Ruminococcus bromii* and *Butyricimonas synergistica* was significantly up-regulated in OVX as compared to Sham group (**Figure 5**). By comparing OVX+ICA to OVX, the phylum Elusimicrobia, an unidentified class in Elusimicrobia, the order Elusimicrobiales, the families Prevotellaceae and Elusimicrobiaceae and the genus *Elusimicrobium* and an unidentified genus in Corynebacteriaceae were significantly up-regulated (**Figure 5**). Moreover, the ovariectomy also resulted in a significant down-regulation of the phylum Actinobacteria, an unidentified class in Actinobacteria, the genera *Lachnoclostridium*, *Negativibacillus*, *Rikenella* and *Harryflintia*, and the species Lachnospiraceae bacterium A2 (**Figure 5**). Furthermore, more significant differential GM was found in OVX+ICA vs OVX and OVX+E2 vs OVX as compared to another two comparisons (**Figure 5**). One information needed to be noted was that some GM such as Streptococcaceae, *Butyricimonas* and *Lactococcus* having a significant up-regulation trend in OVX significantly decreased in OVX+ICA (**Figure 5**). *Globicatella* was another genus belonging to Lactobacillales in Firmicutes like *Lactococcus* that down-regulated in OVX+ICA (**Figure 5**). While Streptococcaceae, Christensenellaceae, *Adlercreutzia* and *Helicobacter rodentium* showed the same down-regulation trend in both OVX+ICA and OVX+E2, only the first one was up-regulated in OVX group (**Figure 5**).

**Figure 6:**
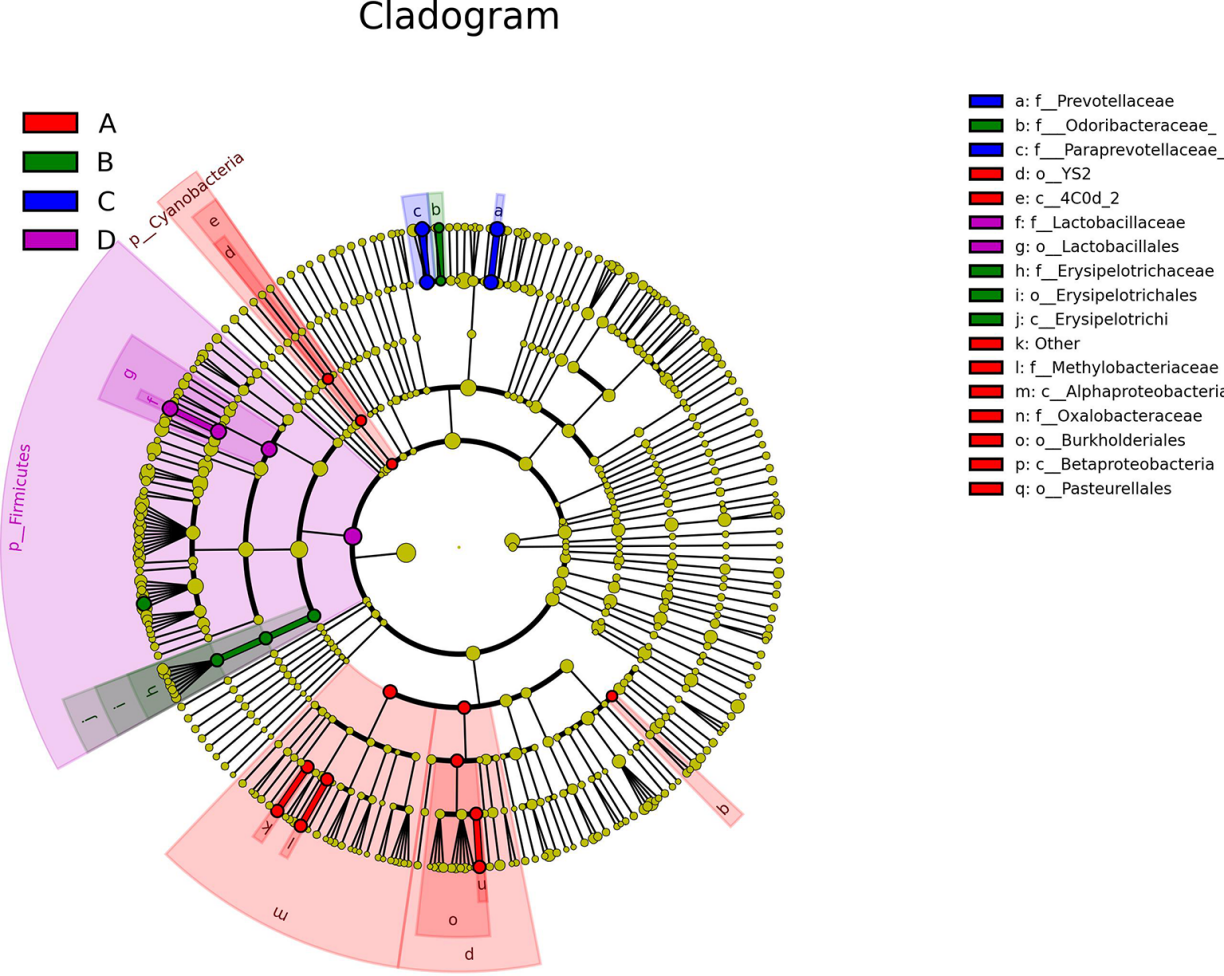
Therapeutic effects of different treatments on the structure and key phylotypes of the GM displayed by cladogram based on LefSe analysis (n = 10). The levels represent, from the inner to outer rings, phylum, class, order, family, genus and species, which can identify the specific community or species that make a significant difference in sample classification. **(A-D)** represent Sham, OVX, OVX+ICA and OVX+E2 groups, respectively.

Although the predominant GM in all treatments belonged to Firmicutes and Bacteroidetes, the key phylotypes making the sample in each treatment distinguishable from others were varied. As shown in **Figure 6** and **Supplementary Figure S2**, the key phylotypes of GM in Sham group showed higher diverse than those observed in other treatments. In OVX group, the genus *Oscillospira* and the family Erysipelotrichaceae in Firmicutes and the genus *Butyricimonas* belonging to Odoribacteraceae in Bacteroidetes were abundant GM (**Figure 6**; **Supplementary Figure S2**). However, the rats administrated with ICA or E2 harbored major GM such as *Paraprevotella* and *Prevotella* in Bacteroidetes or *Lactobacillus* in Firmicutes, respectively (**Figure 6**; **Supplementary Figure S2**). The presence of Erysipelotrichi, Erysipelotrichales, Lactobacillales, Lactobacillaceae, Paraprevotellaceae, Prevotellaceae, *Paraprevotella*, *Prevotella*, *Oscillospira* and *Butyricimonas* was in accordance with the results as shown in **Figure 5**. These data showed that different treatments modulated specific GM, and the diverse GM might co-work to regulate the estrogen homeostasis and bone metabolism.

### Identification of Key Fecal Metabolites Correlated to ICA Administration

Based on metabonomic analysis in POS mode, a total of 15 metabolites were significantly up- or down-regulated between Sham and OVX groups (**Supplementary Table S1**). Among which, 3-Amino-3-(4-Hydroxyphenyl)propanoate, nicotinate, 1-Palmitoylglycerol, Ile-Leu, xanthosine, N-Tigloylglycine, 1-Aminocyclohexanecarboxylic acid, D-Alanyl-D-alanine (D-Ala-D-Ala), L-saccharopine, stearidonic acid, Pro-Asn, triethanolamine and 2’-O-methylcytidine were significantly up-regulated, while N-(omega)-Hydroxyarginine and D-Pipecolinic acid were significantly down-regulated (**Supplementary Table S1**). Compared to OVX, ICA administration resulted in a significant up-regulation of 6 out of 9 differential metabolites, being the highest FC of 6.74 (NG, NG-dimethyl-L-arginine (ADMA); VIP = 2.80; *p <* 0.001) (**Supplementary Table S1**). 1-Aminocyclohexanecarboxylic acid and L-Saccharopine followed similar trend in both Sham vs OVX and OVX+ICA vs OVX (**Supplementary Table S1**). However, most significant differential metabolites between OVX+E2 and OVX were similar to Sham vs OVX and OVX+ICA vs OVX except for Leu-Gly, phenylenthylamine, Thr-Val and Leu-Thr, while only 1-Aminocyclohexanecarboxylic acid was found in all comparisons (**Supplementary Table S1**). The comparison between OVX+E2 and OVX+ICA showed that only D-Ala-D-Ala and ADMA those also observed in OVX+E2 vs OVX or OVX+ICA vs OVX were down- and up-regulated, respectively (**Supplementary Table S1**). A hierarchical clustering heat map showed that the significant differential metabolites in different samples within the same group were similar and varied in different groups (**Supplementary Figure S3A**).

A NEG mode-based analysis also identified several significant differential metabolites in different treatments. Briefly, except for deoxycholic acid and 3-Methyladipic acid, other metabolites including tetrahydrocorticosterone, N-Acetyl-L-phenylalanine, 1-Oleoyl-L-.alpha.-lysophosphatidic acid, lithocholic acid, D-Glucosamine 6-phosphate, succinate, propionic acid, adynerin, 15(S)-15-methyl PGF2alpha, cholic acid, glutaric acid and suberic acid were up-regulated significantly in Sham vs OVX (**Supplementary Table S1**). In OVX+ICA vs OVX, only two metabolites linoleic acid and guanosine were significantly up- and down-regulated, respectively (**Supplementary Table S1**). Unlike the results observed in POS mode, NEG mode only identified 4 metabolites being found in all the three comparisons (**Supplementary Table S1**; **Supplementary Figure S3B**). Interestingly, there were 4 metabolites, i.e., 15(S)-15-methyl PGF2alpha, lithocholic acid, lumichrome and linoleic acid, observed in one or two of the above comparisons showed significant difference between OVX+ICA vs OVX+E2 (**Supplementary Table S1**).

Collectively, ovariectomy, ICA or E2 administration resulted in a considerable change of 66 metabolites (**Supplementary Table S1**), those of which observed in two or three of the treatments might be important in the development or improvement of PMOP. They were tetrahydrocorticosterone, adynerin, 3-Amino-3-(4-Hydroxyphenyl)propanoate, stearidonic acid, triethanolamine, D-Ala-D-Ala, lithocholic acid, 1-Aminocyclohexanecarboxylic acid, L-Saccharopine, suberic acid, ADMA, Arg-Met, lumichronme, linoleic acid and deoxyinosine (**Supplementary Table S1**).

### Unveiling Mechanisms Underlying ICA-Mediated Bone Metabolism Regulation Based on GM-Fecal Metabolite Relationships

**Figure 7:**
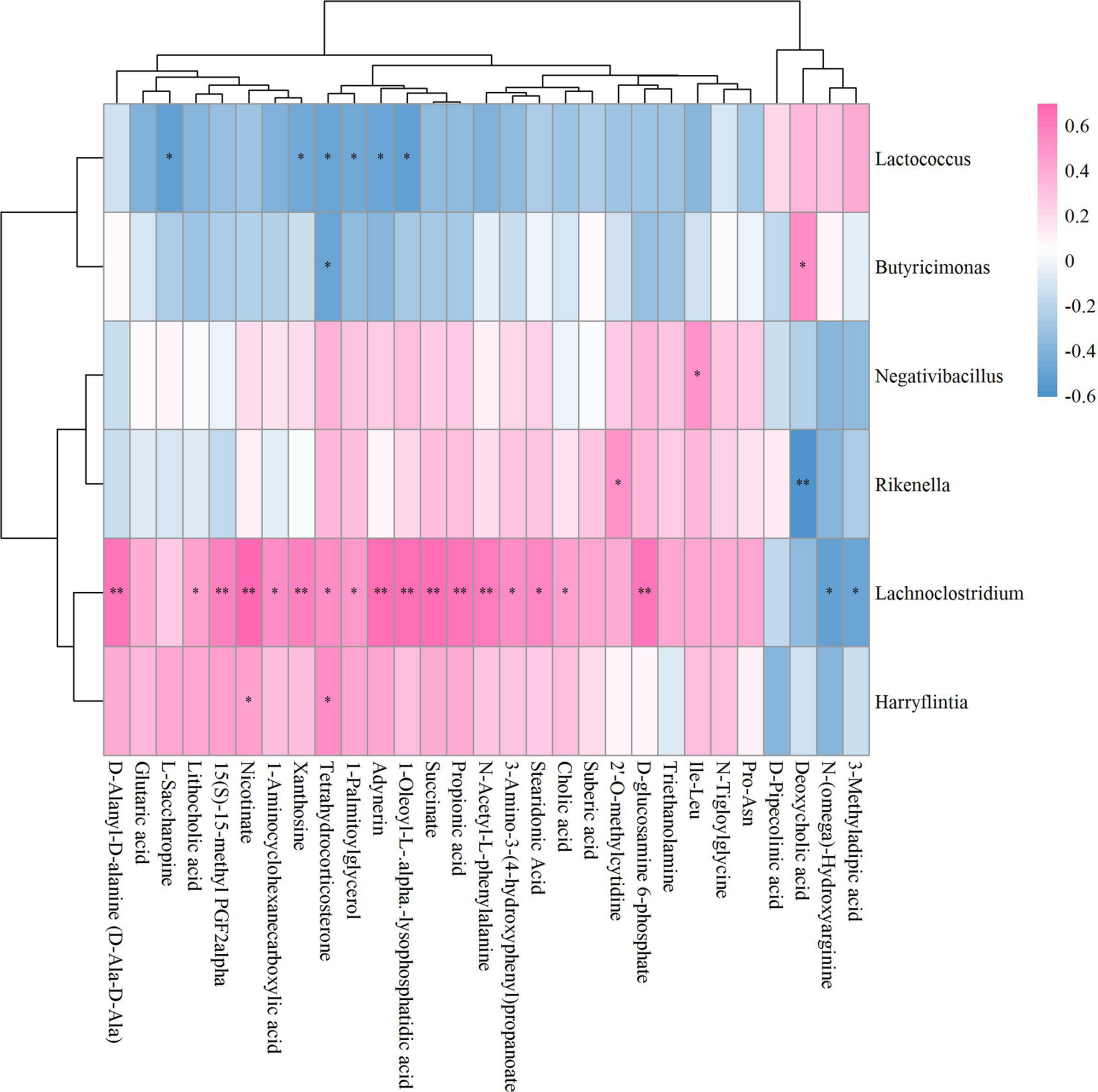
Hierarchical clustering heat map of the significant differential GM and fecal metabolites in Sham and OVX groups based on Spearman correlation analysis (n = 10). * and ** mean a significant correlation between GM and fecal metabolites was found based on *p* < 0.05 and *p* < 0.01, respectively.

The results of the correlation coefficient calculation between significant differential GM and fecal metabolites were given in **Supplementary Table S2**. Six GM in genera *Lactococcus*, *Butyricimonas*, *Negativibacillus*, *Rikenella*, *Lachnoclostridium* and *Harryflintia* were significantly correlated to 23 fecal metabolites in Sham vs OVX (**Figure 7**). Among which, *Lachnoclostridium* correlated significantly to most of the given metabolites, followed by *Lactococcus* and *Rikenella*, but *Butyricimonas*, *Negativibacillus* and *Harryflintia* only significantly correlated to one or two metabolites (**Figure 7**; **Supplementary Table S2A**). The network analysis showed that *Lachnoclostridium*, *Lactococcus*, *Harryflintia*, *Butyricimonas* and *Rikenella* had a higher correlation with 1-Aminocyclohexanecarboxylic acid, 1-Oleoyl-L-.alpha.-lysophosphatidic acid, 15(S)-15-methyl PGF2alpha, 2’-O-methylcytidine, 3-Amino-3-(4-hydroxyphenyl)propanoate, adynerin, D-Ala-D-Ala, D-Glucosamine 6-phosphate, deoxycholic acid, L-Saccharopine, N-(omega)-Hydroxyarginine, N-Acetyl-L-phenylalanine, nicotinate, propionic acid, stearidonic acid, succinate, tetrahydrocorticosterone and xanthosine (**Supplementary Figure S4**; **Supplementary Table S2A**).

**Figure 8:**
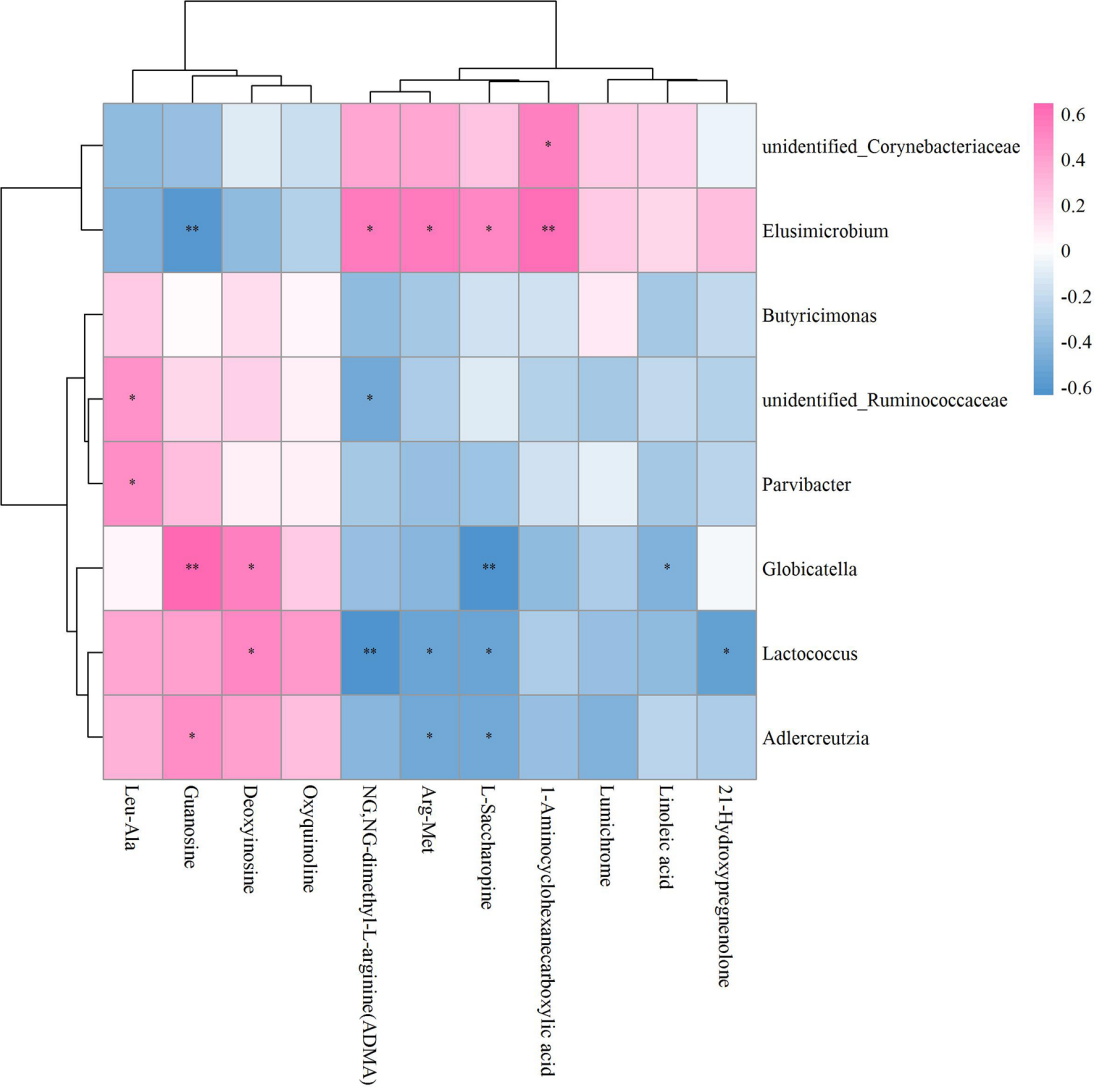
Hierarchical clustering heat map of the significant differential GM and fecal metabolites in OVX+ICA and OVX groups based on Spearman correlation analysis (n = 10). * and ** mean a significant correlation between GM and fecal metabolites was found based on *p* < 0.05 and *p* < 0.01, respectively.

As to OVX+ICA and OVX, the significant correlation was found between the GM such as unidentified_Corynebacteriaceae, *Elusimicrobium*, unidentified_Ruminococcaceae, *Parvibacter*, *Globicatella*, *Lactococcus* and *Adlercreutzia* and the metabolites such as Leu-ALA, guanosine, deoxyinosine, ADMA, Arg-Met, L-Saccharopine, 1-Aminocyclohexanecarboxylic acid, linoleic acid and 21-Hydroxypregnenolone (**Figure 8**). The key GM were *Elusimicrobium*, *Lactococcus* and *Globicatella*, which showed high correlation with ADMA, guanosine, 1-Aminocyclohexanecarboxylic acid, Arg-Met, 21-Hydroxypregnenolone, deoxyinosine and L-Saccharopine (**Supplementary Figure S5**; **Supplementary Table S2B**).

**Figure 9:**
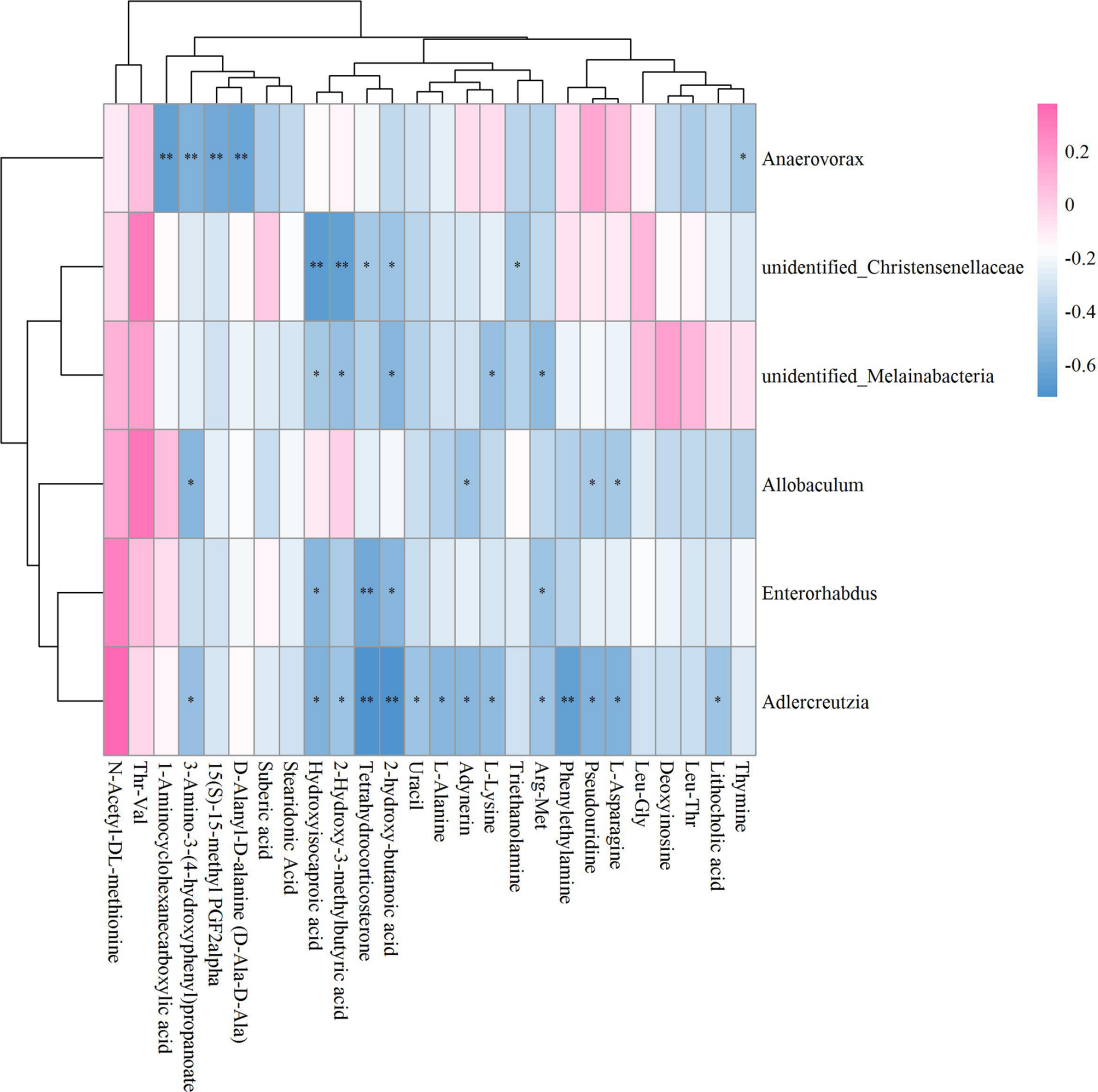
Hierarchical clustering heat map of the significant differential GM and fecal metabolites in OVX+E2 and OVX groups based on Spearman correlation analysis (n = 10). * and ** mean a significant correlation between GM and fecal metabolites was found based on *p* < 0.05 and *p* < 0.01, respectively

**Figure 10:**
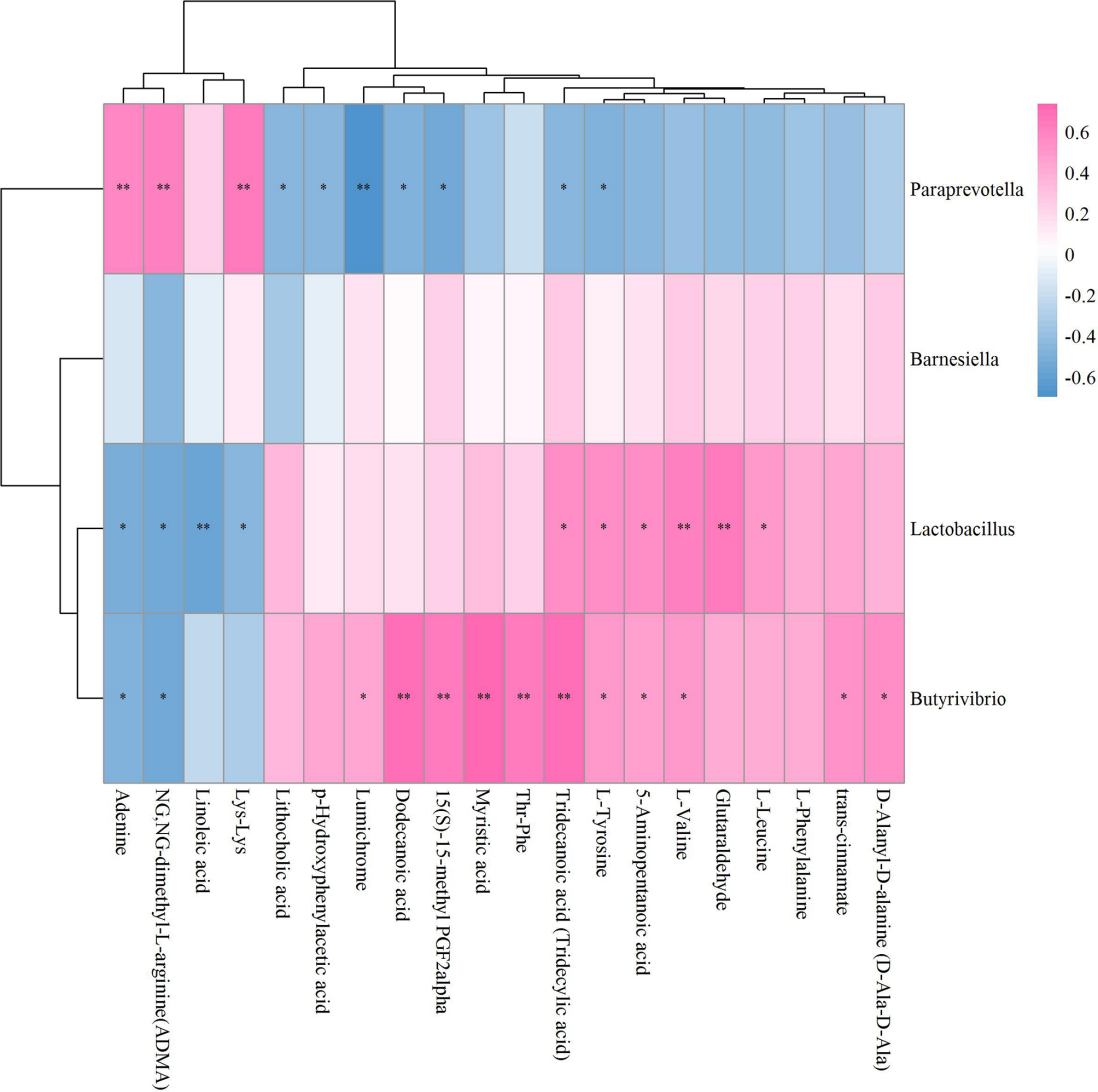
Hierarchical clustering heat map of the significant differential GM and fecal metabolites in OVX+ICA and OVX+E2 groups based on Spearman correlation analysis (n = 10). * and ** mean a significant correlation between GM and fecal metabolites was found based on *p* < 0.05 and *p* < 0.01, respectively.

Unlike Sham vs OVX and OVX+ICA vs OVX, no positive correlation with statistical significance between GM and fecal metabolites in OVX+E2 vs OVX was found (**Figure 9**). Most of the key metabolites were correlated to at least one of the significant differential GM such as *Anaerovorax*, unidentified_Christensenellaceae, unidentified_Melainabacteria, *Allobaculum*, *Enterorhabdus* and *Adlercrutzia* (**Figure 9**; **Supplementary Table S2C**). The most eye-catching GM was *Adlercreutzia*, also observed in OVX+ICA, that correlated to most of the key metabolites (**Supplementary Figure S6**). Finally, we analyzed the correlation of GM and fecal metabolites between OVX+ICA and OVX+E2. The results showed that *Paraprevotella*, *Lactobacillus* and *Butyrivibrio* were significantly correlated to most of the significant differential metabolites except for L-Phenylalanine (**Figure 10**; **Supplementary Table S2D**).

## Discussion

In this study, ICA with an estrogen-like function improved the bone micro-architecture and the serum biomarkers associated with bone metabolism in OVX rats. Different treatments modulated specific GM and regulated fecal metabolite alterations. Several key GM were correlated to bile acid, amino acid and fatty acid, suggesting their potential roles in regulating the bone homeostasis. Such metabolites significantly correlated to the significant differential GM could be probably the targets to further understand the underlying metabolisms in regulating PMOP. These findings have provided new evidence for using ICA as a potential therapy for PMOP at the level of GM-bone axis.

### Verification of *In Vivo* Therapeutic Effects of ICA on Inhibiting Bone Loss

The estrogen deficiency-induced PMOP has a typical symptom of bone metabolic disorder, which can stimulate RANKL production to prolong the life span of osteoclasts and reduce the life span of osteoblasts (35). Hormone replacement therapy is often used to prevent or ameliorate bone loss by promoting osteocalcin gene expression (4). The use of E2 in prevention and treatment of PMOP has been widely used for a long time (36, 37). Recent study further verified that E2 administration was able to increase BMD and BV/TV probably by changing miRNAs expression (5). As an alternative candidate for estrogen replacement due to its structural similarity with E2, ICA has shown outstanding effects on the improvement of PMOP (9). Here, we found a great ability of ICA administration to improve the micro-architecture of bone trabecula and increase BMD and BV/TV in OVX rats (**Figure 1**), in accordance with the recent studies from Wei et al. (14) and Zhou et al. (16). A significant decrease of BGP and RANK and increase of OPG in OVX rat serum suggested a direct evidence of ICA-induced osteogenic differentiation (38), which was probably mediated *via* Wnt/β-catenin pathway like other TCMs such as *radix Salviae miltiorrhizae* (RSM) and Liuwei Dihuang (10, 13, 39, 40). Other signaling pathways including IGF-I coupled non-genomic ERα and STAT associated with ICA-induced OP improvement have also been documented (16, 17), but whether the pathways RANKL/RANK/TRAF6 and OPG/RANKL/cathepsin K identified in other TCMs (39, 41) can be activated by ICA still warrants further investigations. As to another biomarker of bone sorption, TRACP can also be up-regulated following ovariectomy and decreased to a normal level of control (38). It is noting that the decrease of adipocytes also indicates a positive effect of ICA on osteogenic differentiation, which is probably due to the inhibition of AMPK/mTOR signaling pathway (42, 43) or the activation of RhoA-TAZ signaling pathway (44). Apparently, similar to E2 or other reported TCMs, ICA has played an equal role in improving bone metabolisms through regulating serum BGP, RANK, RANKL, OPG and TRACP concentrations.

### Verification of GM-Related Targets Towards PMOP Regulation by High-Throughput Sequencing and Metabolomic Analyses

Gut microbes are involved in the regulation of bone homeostasis mainly *via* intestinal barrier and nutrient absorption (involving SCFAs), immunoregulation (Th-17 and T-reg cells balance) and regulation of intestinal-brain axis (involving 5-HT) (19). Several studies have found a higher abundance of Firmicutes and lower abundance of Bacteroidetes in OVX rats than Sham group (20, 25). In our study, ICA administration reversed the abundances of Firmicutes and Bacteroidetes (**Figure 4A**), in accordance with the findings from Xiong et al. (25) and Hong et al. (20) although the microbial compositions in genus level were different (**Figure 4B**). In fact, several key genera such as *Prevotella*, *Barnesiella*, *Lactococcus*, *Streptococcus* and *Ruminococcus* correlating to serum indexes of bone homeostasis and inflammation (TNF-α and IL-17) have been reported (21). Here, we detected a significant correlation of *Ruminococcus* with RANK, OPG and TRACP (**Table 1**) as reported by Ma et al. (45), implying that ICA can modulate GM to influence serum indexes to regulate bone metabolisms. Moreover, ICA administration-induced improvement of intestinal barrier function has been proven to be associated with GM by inhibiting NF-κB or p38 MAPK signaling pathway (25, 26). Combining our results with those from other studies suggest that both the inhibition of NF-κB pathway to reduce bone resorption and the activation of Wnt/β-catenin pathway to enhance bone formation after E2 analogues administration may be associated with GM (21, 46). Therefore, further investigations on the effects of ICA on the prevention and treatment of PMOP can be performed by determining the inflammatory factors and correlating the data to GM and serum indexes.

Different treatments modulate specific microbial communities, *t*-test is able to identify the significant differential GM (47). By emphasizing on the cases for OVX and OVX+ICA, 3 GM with significant difference were identified (**Figure 5**). Except for *Butyricimonas*, both Streptococcaceae and *Lactococcus* are belonging to Firmicutes. Although Streptococcaceae, *Lactococcus* and *Butyricimonas* were not significantly correlated to serum indexes (**Table 1**), the first two were reported to have close relationships with IL-17, TNF-α and Wnt1 according to Li J. et al. (21). In contrast, other GM correlated well to serum indexes have not shown significant difference between treatments (**Figure 5**; **Table 1**). In other words, the high abundant and significant different GM among treatments likely involved in different processes to regulate bone metabolism due to the complex mechanisms as previously described (19). Interestingly, Streptococcaceae, Christensenellaceae, *Adlercreutzia* and *Helicobacter rodentium* followed similar trend in OVX+ICA vs OVX and OVX+E2 vs OVX, again verifying the potential roles of ICA, as an analogue of E2, in the prevention and treatment of PMOP (9). Since LefSe analysis and LDA obtain unique GM in one group apart from others, they provide more information about the key phylotypes in each treatment (20, 33). In summary, the significant differential GM between treatments and the key phylotypes in each treatment suggest a full picture of the interactions between ICA and GM.

Besides the modulation towards GM, different treatments also resulted in a considerable change of the fecal metabolites (**Supplementary Table S1**). Bile acid is an important compound which has been reported to have vital roles in bone metabolism (48, 49). The primary bile acid (e.g., cholic acid) is synthesized in liver and modified to secondary products such as lithocholic acid, deoxycholic acid and their conjugated species in gut by microbes, finally enters portal veins (50). The hepatoenteral circulation maintains the stability of bile acid metabolism, thus both the serum and fecal bile acids have been used to evaluate their roles in bone metabolisms (51, 52). In our study, the ovariectomy enhanced cholic acid and lithocholic acid assimilation but promoted deoxycholic acid loss, which was likely attributed to the PMOP development as reported previously (52, 53). However, the regulation of bile acid metabolism might be one of the possible mechanisms to improve PMOP by E2 but not ICA administration (**Supplementary Table S1**). It has shown that the overconsumption of *n*-6 polyunsaturated fatty acids (PUFA) may result in a high *n*-6 to *n*-3 ration and thus lead to an increased pathogenesis of PMOP by promoting low-grade chronic inflammation (LGCI) (54). The anti-inflammatory role of stearidonic acid (a *n*-3 PUFA derived from linoleic acid) in improving PMOP have been widely studied (54), which was also supported by our data that E2 administration promoted a significant increase of stearidonic acid (FC = 1.49, *p <* 0.001). It was interesting to note that ICA administration also significantly up-regulated the precursor of stearidonic acid, i.e., linoleic acid, by FC of 1.36 (*p <* 0.05), which was 1.32 times of E2 group (*p <* 0.05). The relationships between linoleic acid and bone biology have been widely studied and recently verified to promote bone formation in OVX rats by co-working with ultraviolet B treatment (55, 56). L-Saccharopine is an important metabolite in amino acid metabolism and often decrease during the progression of OP and PMOP (57, 58). While ovariectomy resulted in a significant decrease of L-Saccharopine (FC = 0.69, *p* < 0.05), the ICA administration significantly up-regulated it by FC of 1.47 (*p*< 0.05), similar to the effects of velvet collagen hydrolysate (57) and sialoglycoprotein (58). Since its vital roles in amino acid metabolism and PMOP improvement, we conclude that L-Saccharopine can be an important biomarker to further evaluate the mechanisms of ICA towards PMOP prevention and treatment. In our study, we also noted a significant alteration of 1-Aminocyclohexanecarboxylic acid in OVX as well as the ICA and E2 administrated rats (**Supplementary Table S1**). 1-Aminocyclohexanecarboxylic acid is an antistaphylococcal amino acid and has been used to prepare 4-Aminophenoxyacetic acid, a novel class of reversible cathepsin K inhibitors (59–61). However, whether ICA and E2, similar to RSM, possesses the ability to improve PMOP *via* OPG/RANKL/cathepsin K signaling pathway with the aid of 1-Aminocyclohexanecarboxylic acid warrants further study (39).

Other metabolites involved in the regulation of PMOP when administrated with E2 were also found in this study (**Supplementary Table S1**). Unlike typical glucocorticoids, which have systematic side effects such as OP, abdominal obesity and glaucoma, tetrahydrocorticosterone has shown outstanding potentials in defensing inflammation with fewer side effects (62). Here, we found a down-regulation of tetrahydrocorticosterone by 40 times in OVX rats as compared to Sham group (*p <* 0.001), while E2 significantly increased its production (FC = 1.82, *p <* 0.001). Several studies have also shown the relationship between 2-Hydroxy-butanoic acid and TRACP (63), the effects of L-Lysine on stimulating the osteoblast proliferation and synthetic activity (64), the transformation between L-Alanine and tryptophane and its roles in regulating PMOP (65), and the prevention of bone loss by enhancing L-Asparagine production and OPG increase (66, 67). It seemed that, however, ICA-induced improvement of PMOP cannot be associated with the above-mentioned metabolites although no significant difference between ICA and E2 treatments were observed (**Supplementary Table S1**). These results showed that ICA had different mechanisms for the treatment of PMOP as compared to E2 administration.

### Exploration of Potential GM-Bone Axis Mechanisms Underlying Therapeutic Effects of ICA on PMOP

It has well-documented that the gut microbial alterations can impair bone strength and tissue material properties (68). *Lachnoclostridium* is an abundant GM having key correlations with bone mass (69, 70), which was supported by our results that this genus significantly correlated to most of the significant differential metabolites (**Figure 7**). While *Butyricimonas* can regulate bile acid by influencing vitamin D absorption (24), no study has so far shown the role of *Rikenella* in bile acid metabolism. As to *Adlercreuztia*, it can function in bone metabolism by regulating cholic acid, exactly lithocholic acid in this study, like *Gordonibacter* and *Parvibacter*, which belong to the same family of *Adlercreutzia*, Eggerthellaceae (52). Our data showed that the down- and up-regulation of L-Saccharopine, a lysine degradation intermediate (71), in OVX and ICA groups were associated with *Lactococcus*, as well as the genera *Elusimicrobium*, *Globicatella* and *Adlercreutzia* (**Figure 7**). Other amino acids such as 1-Aminocyclohexanecarboxylic acid, L-Asparagine, L-Lysine, L-Alanine and 2-Hydroxy-butanoic acid significantly correlated to *Lachnoclostridium*, *Elusimicrobium*, *Anaerovorax*, *Adlercreutzia*, *Allobaculum* and *Enterorhabdus*, which were in line with the finding that the association of GM with PMOP was mediated by amino acid metabolism (72). More and more studies have verified that several lactic acid bacteria are capable of the biohydrogenation of linoleic acid (73). Recent study also revealed the prevention of PMOP in OVX rats by *Saururus chinensis* extract due to the involvement of linoleic acid (22). In our study, the GM significantly correlated to metabolites were belonging to two independent families Aerococcacease (*Globicatella*) and Lactobacillaceae (*Lactobacillus*) in the same order Lactobacillales of Firmicutes (**Figures 8**, **10**). Of which, *Lactobacillus* has been widely used to prevent PMOP in OVX model (18, 74, 75). As indicated, the IGF-I signaling pathway was involved in this process (18).

**Figure 11:**
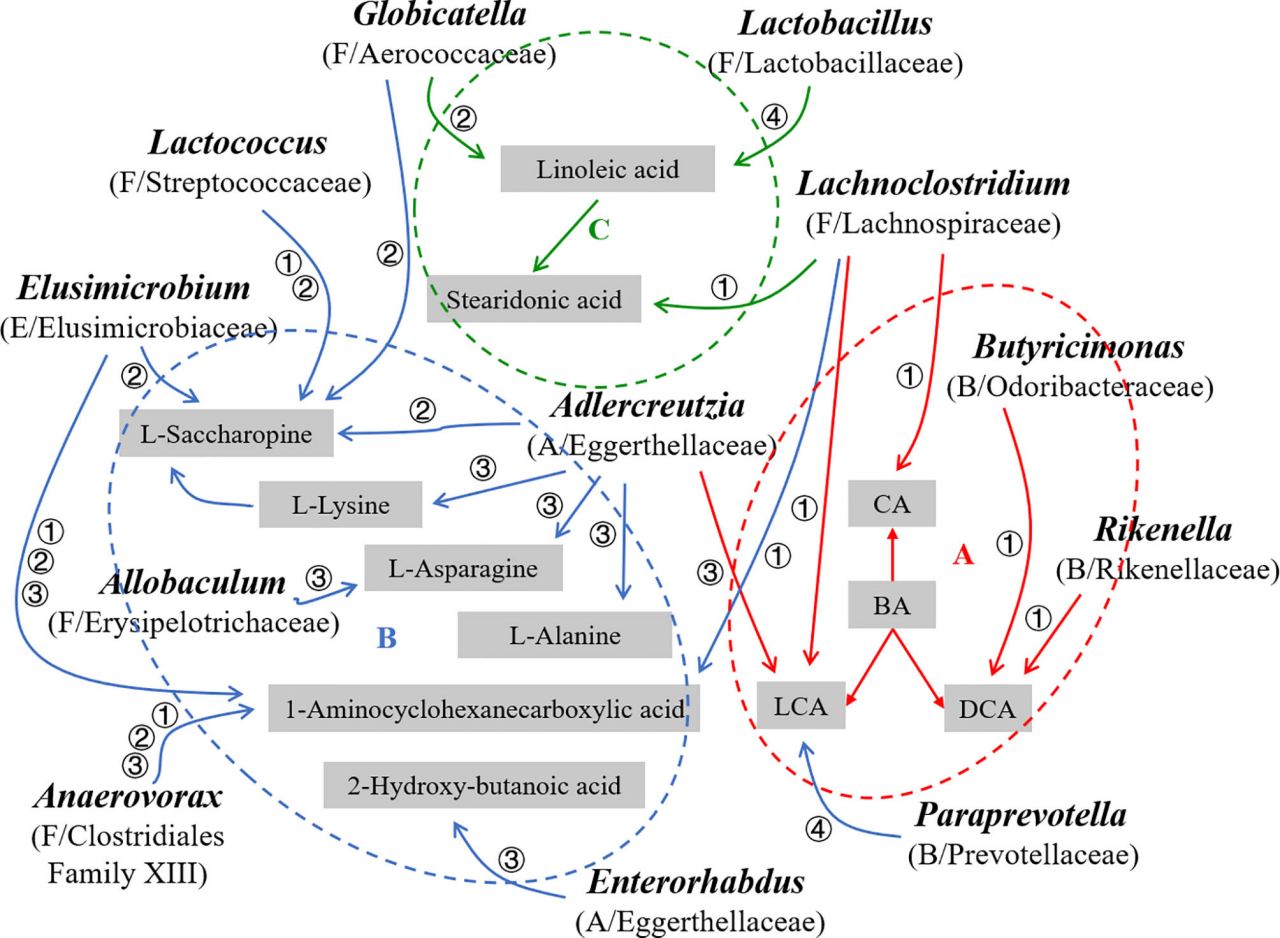
Schematic diagram of the GM and key metabolites probably associated with PMOP development and (or) improvement in OVX rats (n = 10). The red (→, A, ⚬), blue (→, B, ⚬) and green (→, C, ⚬) information represent bile acid, amino acid and fatty acid metabolisms, respectively. A, Actinobacteria; B, Bacteroidetes; F, Firmicutes; E. Elusimicrobia; BA, Bile acid, CA, Cholic acid, LCA, Lithocholic acid, DCA, Deoxycholic acid; ①, ②, ③ and ④ mean Sham vs OVX, OVX+ICA vs OVX, OVX+E2 vs OVX and OVX+ICA vs OVX+E2, respectively.

Taken together, the ovariectomy mainly disrupted bile acid metabolism to induce PMOP (**Figure 11A**), while ICA administration restored the bone metabolism homeostasis through amino acid and fatty acid metabolism (**Figure 11B**), and E2 intervention treatment activated both bile acid and amino acid metabolisms to improve PMOP (**Figure 11C**). We concluded that the improvement of PMOP after ICA or E2 administration was probably in part achieved by GM-mediated bile acid, amino acid and fatty acid metabolisms.

## Conclusions

In this study, ICA exhibited great ability in protecting bone by regulating GM-bone axis in OVX rats. Both GM modulation and fecal metabolite alteration were induced by ICA administration to improve PMOP. The underlying mechanisms included GM-regulated changes of the serum biomarkers RANK, RANKL, OPG and TRACP, and alterations of the fecal metabolites such as bile acid, amino acid and fatty acid. Our data provided evidence for ICA as a novel potential therapy for PMOP, and shed lights onto the PMOP pathogenesis from a new perspective.

## Data Availability Statement

The original contributions presented in the study are publicly available. This data can be found here: NCBI, PRJNA807063.

## Ethics Statement

The animal study was reviewed and approved by The Laboratory Animal Welfare and Ethics Committee of Fujian University of Traditional Chinese Medicine.

## Author Contributions

Conceptualization, XL. Data Curation, SSW, SJW, XW, YX, and XL. Funding acquisition, SSW and XL. Sources, XL. Investigation, SJW and YX. Methodology, SJW, XW, YX, XZ, YH, LW, HZY, and XL. Supervision, HZY and XL. Writing—original draft, SSW and XL. Writing—review and editing, SSW, SJW, XW, YX, XZ, YH, HY, LL, LW, HZY, and XL. All authors contributed to the article and approved the submitted version.

## Funding

This work was funded by the National Natural Science Foundation of China (No. 82074461), the Natural Science Foundation of Fujian Province (No. 2021J01364), the Chen Keji Development Fund of Integrative Medicine (No. 2020004) and the Research Start-up Fund of Fujian University of Traditional Chinese Medicine (No. X2020011-Talent).

## Conflict of Interest

The authors declare that the research was coducted in the absence of any commercial or financial relationships that could be construed as a potential conflict of interest.

## Publisher’s Note

All claims expressed in this article are solely those of the authors and do not necessarily represent those of their affiliated organizations, or those of the publisher, the editors and the reviewers. Any product that may be evaluated in this article, or claim that may be made by its manufacturer, is not guaranteed or endorsed by the publisher.
