# Supplementary material for: Effects of Icariin on Modulating Gut Microbiota and Regulating Metabolite Alterations to Prevent Bone Loss in Ovariectomized Rat Model
Source: Front Endocrinol (Lausanne). 2022 Mar 24;13:874849. doi: 10.3389/fendo.2022.874849 (PMC8988140; doi:10.3389/fendo.2022.874849)
Supplement: Supplementary file 1 [file Image_1.pdf]

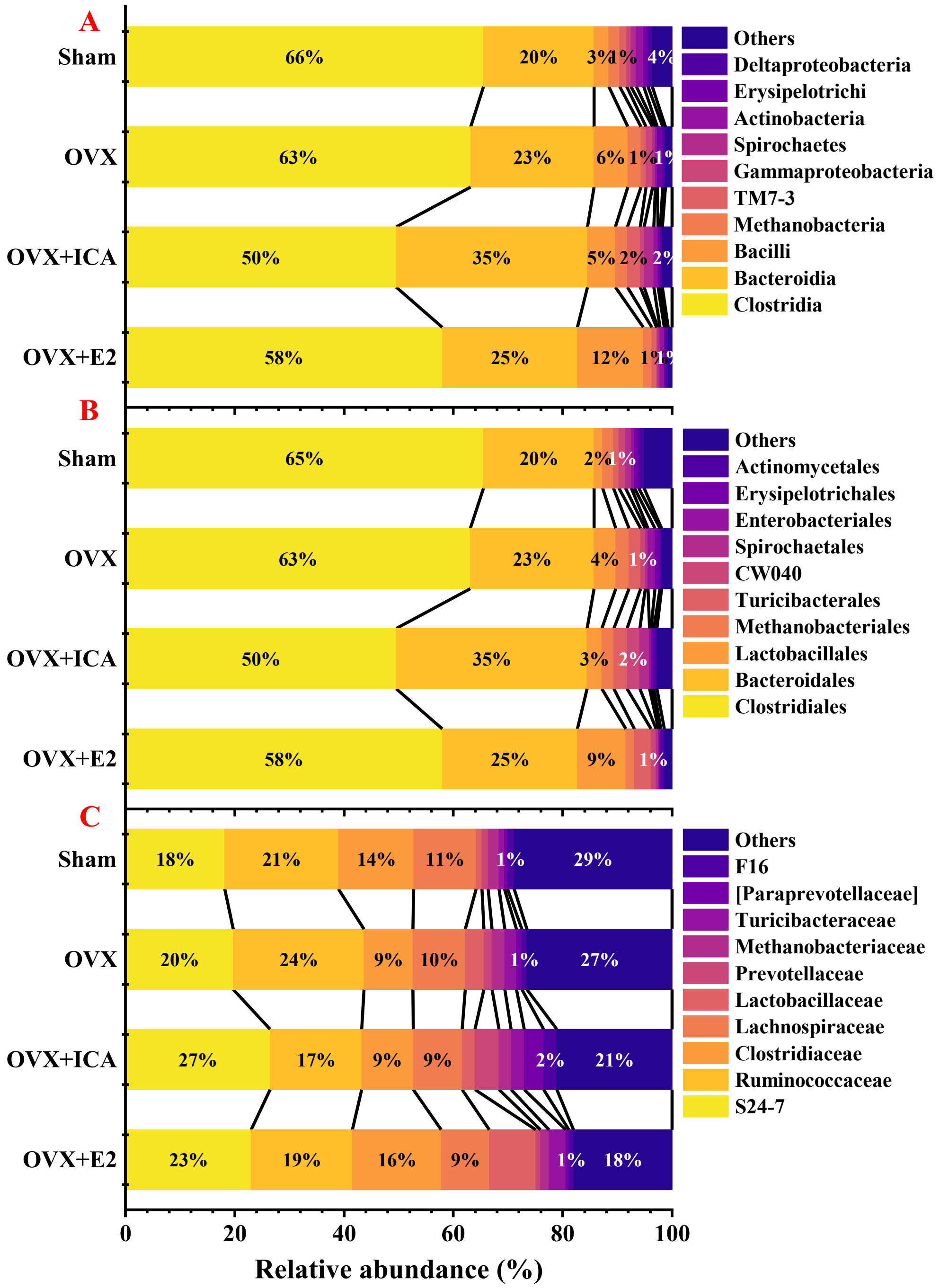

Figure S1. Therapeutic effects of different treatments on the relative abundance of GM at the taxonomic levels of Class (A), Order (B) and Family (C). Only the Latin manes of the top 10 GM were given, while the low abundant ones were put together and shown as "Others".
