# Supplementary material for: Effects of Icariin on Modulating Gut Microbiota and Regulating Metabolite Alterations to Prevent Bone Loss in Ovariectomized Rat Model
Source: Front Endocrinol (Lausanne). 2022 Mar 24;13:874849. doi: 10.3389/fendo.2022.874849 (PMC8988140; doi:10.3389/fendo.2022.874849)
Supplement: Supplementary file 2 [file Image_2.pdf]

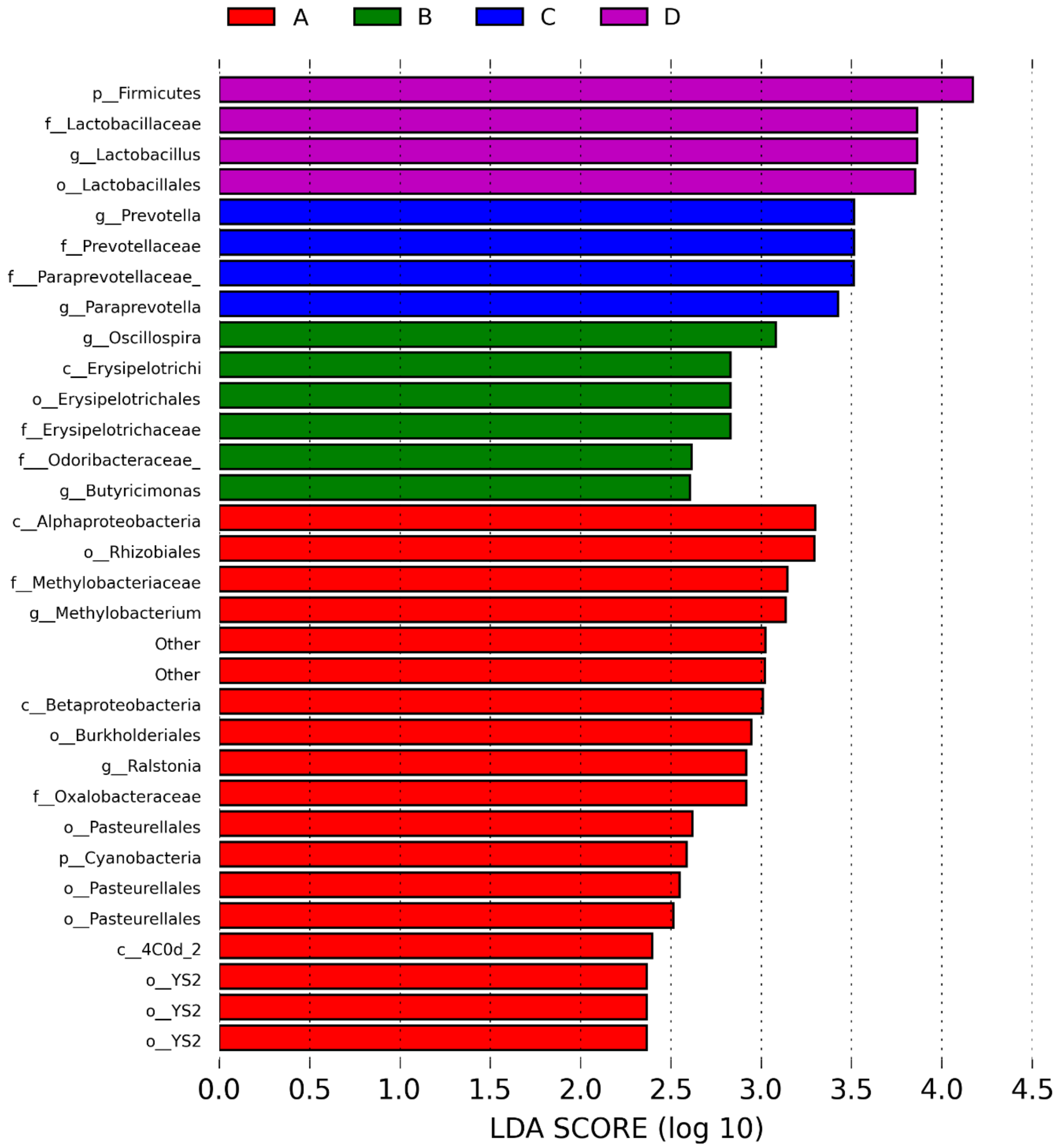

Figure S2. Therapeutic effects of different treatments on the key phylotypes of GM displayed by histogram based on LDA value. A, B, C and D represent Sham, OVX, OVX+ICA and OVX+E2 groups, respectively.
