# Supplementary material for: Effects of Icariin on Modulating Gut Microbiota and Regulating Metabolite Alterations to Prevent Bone Loss in Ovariectomized Rat Model
Source: Front Endocrinol (Lausanne). 2022 Mar 24;13:874849. doi: 10.3389/fendo.2022.874849 (PMC8988140; doi:10.3389/fendo.2022.874849)
Supplement: Supplementary file 3 [file Image_3.pdf]

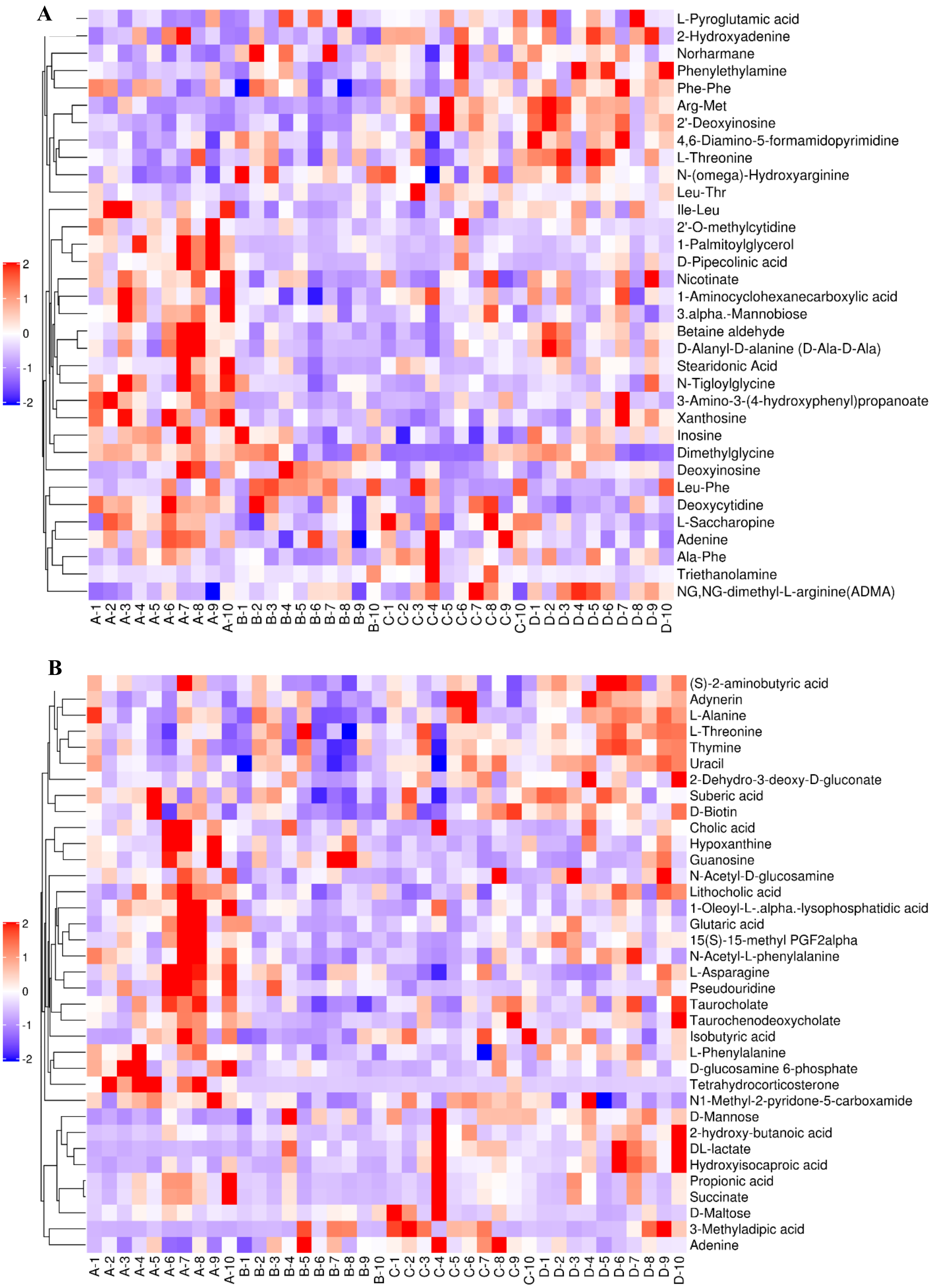

Figure S3. Hierarchical clustering heat map of the significant differential metabolites revealed by POS (A) and NEG (B) modes.
