# Supplementary material for: Effects of Icariin on Modulating Gut Microbiota and Regulating Metabolite Alterations to Prevent Bone Loss in Ovariectomized Rat Model
Source: Front Endocrinol (Lausanne). 2022 Mar 24;13:874849. doi: 10.3389/fendo.2022.874849 (PMC8988140; doi:10.3389/fendo.2022.874849)
Supplement: Supplementary file 7 [file Image_7.pdf]

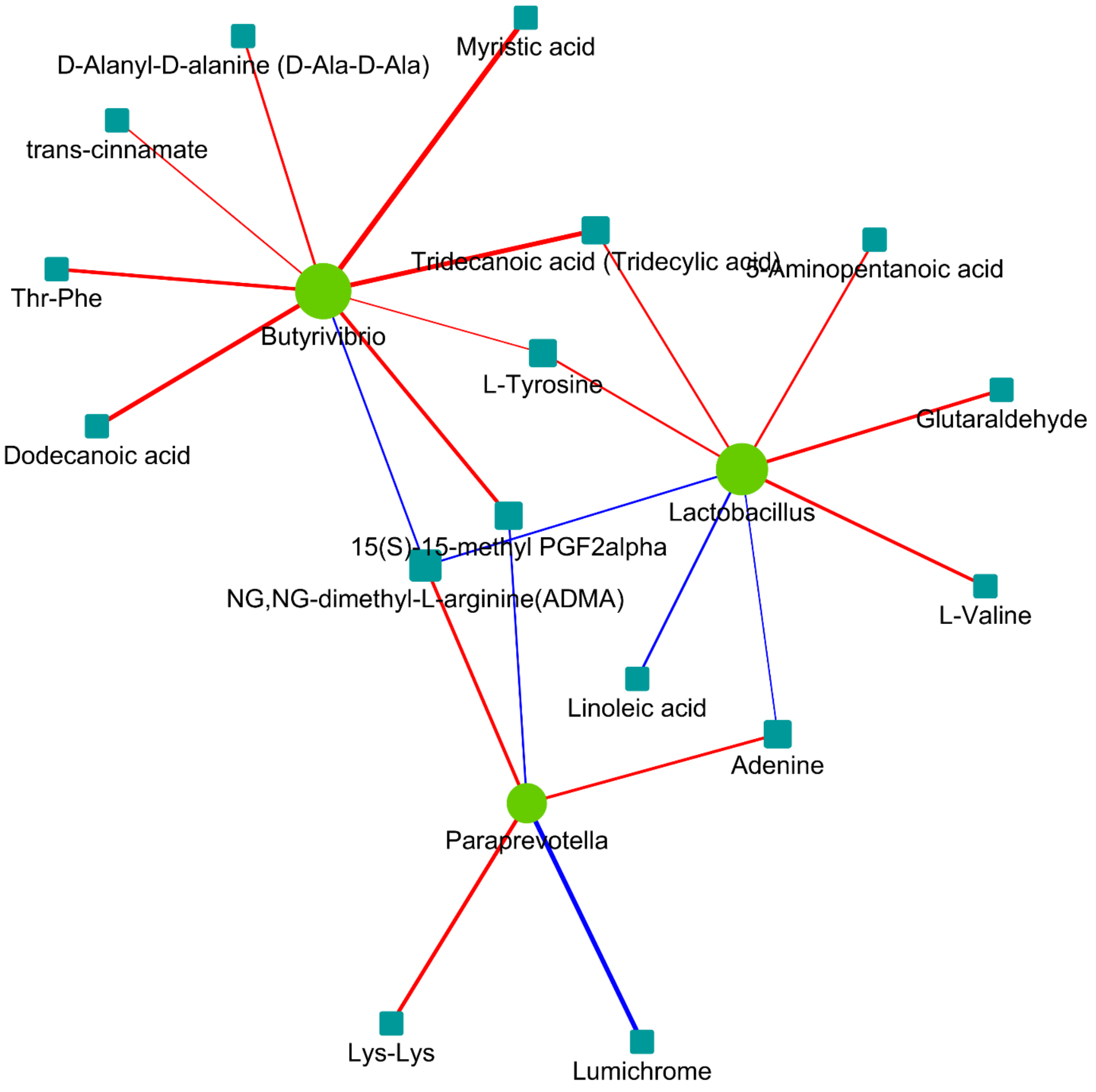

Figure S7. Spearman correlation analysis-based network of the significant differential GM and fecal metabolites in OVX+ICA and OVX+E2 groups. Only the GM and fecal metabolites with an absolute value of correlation coefficient within [0.5, 1] and p-value < 0.05 were listed.
